# The Evolution of SHARE: An evidence-based program for persons with early-stage dementia and their care partners

**DOI:** 10.1093/geroni/igaf122.2533

**Published:** 2025-12-31

**Authors:** Zoe Fete, Silvia Orsulic-Jeras, Donna Salaam, Megan Huth, Sara Powers

**Affiliations:** Benjamin Rose, Cleveland, Ohio, United States; Benjamin Rose, Cleveland, Ohio, United States; Benjamin Rose, Cleveland, Ohio, United States; Benjamin Rose Institute on Aging, Cleveland, Ohio, United States; Benjamin Rose, Cleveland, Ohio, United States

## Abstract

Best Programs for Caregiving (BPC) is an online database of nearly 50 evidence-based dementia caregiving programs. One such program is SHARE for Dementia, which targets care values/preferences and planning for the future, including both the person living with dementia and their care partner. Beginning in 1997 with a series of pre-intervention studies, SHARE has since gone through multiple adaptations and technological developments. In 2023, the BPC team collected online surveys from organizations (n = 8) delivering SHARE across the US about their implementation. In reviewing data, all eight organizations (100%) indicated SHARE was being delivered in-person, with two of those organizations also indicating an online component. Only one organization indicated they were delivering the program at a national level (e.g., any care dyad is eligible no matter location), with the other seven organizations offering the program locally or statewide. Results also indicated that at the time of data collection SHARE implementation sites did not identify specific diverse communities they were tailoring the delivery of the program for, including things such as adapting marketing materials and specific staff trainings. Discussion will focus on improvements to the program’s ability to be offered virtually (e.g., creating a web-based application), improving accessibility to the program for dementia care dyads, as well as increasing the feasibility to implement among community-based organizations. Additionally, to better meet the needs of diverse communities, details surrounding the current development of a cultural adaptation of SHARE for Black and African American families impacted by dementia will be discussed.

